# Draft Genome Sequences of Five Diverse *Klebsiella* Species Isolates from Intensive Care Unit Patients

**DOI:** 10.1128/MRA.01131-20

**Published:** 2020-11-19

**Authors:** Tracy H. Hazen, Stephanie Hitchcock, Lyndsay M. O’Hara, Jane M. Michalski, J. Kristie Johnson, David P. Calfee, Loren G. Miller, Anthony D. Harris, David A. Rasko

**Affiliations:** a Institute for Genome Sciences, University of Maryland School of Medicine, Baltimore, Maryland, USA; b Department of Microbiology and Immunology, University of Maryland School of Medicine, Baltimore, Maryland, USA; c Department of Pathology, University of Maryland School of Medicine, Baltimore, Maryland, USA; d Division of Infectious Diseases, Weill Cornell Medicine, New York, New York, USA; e LA Biomedical Research Center at Harbor-UCLA Medical Center, Torrance, California, USA; f Department of Epidemiology and Public Health, University of Maryland School of Medicine, Baltimore, Maryland, USA; Loyola University Chicago

## Abstract

We have examined the draft genomes of five isolates from two different *Klebsiella* species obtained from intensive care unit patients at two geographically distributed hospitals to examine the genomic diversity of hospital-acquired organisms in this understudied population.

## Abstract (precis)

We have examined the draft genomes of five isolates from two different *Klebsiella* species obtained from intensive care unit patients at two geographically distributed hospitals to examine the genomic diversity of hospital acquired organisms in this understudied population.

## ANNOUNCEMENT

This study was undertaken to examine the genomic variation of multiple species of isolates obtained from perirectal samples from patients in the intensive care unit (ICU). Patients who were admitted to an ICU at one of two hospitals—one in Baltimore, MD, and one in Torrance, CA—between September 2017 and April 2019 were enrolled in the study. Samples were collected under University of Maryland School of Medicine protocol HP-00066759. These patients had a prior surveillance or clinical Klebsiella pneumoniae culture within 7 days of enrollment in a study design similar to that described by O’Hara et al. (1). Each swab was cultured overnight on CHROMagar KPC to identify K. pneumoniae and other *Klebsiella* species (2). Antibiotic susceptibility testing was performed for all single-colony selected isolates by disk diffusion and interpreted in accordance with CLSI guidelines (3). A total of five isolates were examined by genome sequencing in this study, four isolates of Klebsiella variicola and one isolate of Klebsiella quasipneumoniae.

Genomic DNA was isolated from cultures grown in lysogeny broth overnight at 37°C with shaking. DNA was extracted in a 96-well format from 100 μl of sample using the MagAttract PowerMicrobiome DNA/RNA kit (Qiagen, Hilden, Germany) automated on a Hamilton Microlab STAR robotic platform. Bead disruption was conducted on a TissueLyser II (20 Hz for 20 min) instrument in a 96-deep-well plate in the presence of 200 μl phenol-chloroform. Genomic DNA was eluted in 90 μl of water after magnetic bead cleanup. The resulting genomic DNA was quantified with PicoGreen. The sequencing libraries were generated with the KAPA HyperPrep kit (catalog number KK8504) and sequenced on the Illumina NovaSeq 6000 system using a 150-bp × 2 paired-end kit.

The total numbers of reads and bases and the genome coverage generated for each isolate are listed in Table 1. All software was used with default values. Raw sequencing reads were filtered to remove contaminating phiX reads using BBDuk of the BBTools software suite (https://sourceforge.net/projects/bbmap/). The raw reads were also filtered to remove contaminating Illumina adaptor sequences and quality trimmed using Trimmomatic v.0.36 (4). The resulting reads were assembled using SPAdes v.3.13.0 (5). The assemblies were filtered to contain only contigs longer than 500 bp with a k-mer coverage of ≥5×. Genomes containing greater than 500 contigs or an aberrant GC content were removed from further analysis. Relevant statistics (contig number, genome size, GC content, and *N*_50_ value), including GenBank accession and SRA links for each genome assembly, are included in Table 1. The genomes were annotated with PGAP v.4.12 (6). Further analysis will reveal the genome dynamics of these important species in the health care setting, as well as genetic determinants of transmission via health care worker interactions.

**TABLE 1:** Genome metrics

Isolate	Species	Hospital	No. of reads	No. of bases sequenced	Genome coverage (×)	No. of contigs	Genome size (bp)	% GC	*N*_50_ (bp)	GenBank accession no.	SRA accession no.
CRE135	Klebsiella variicola	University of Maryland Medical Center	2,751,678	415,503,378	71	64	5,854,485	57.13	272,686	JABKRN000000000	SRR11819346
CRE165	Klebsiella variicola	University of Maryland Medical Center	3,717,514	561,344,614	103	53	5,448,709	57.52	185,995	JABKRM000000000	SRR11819345
CRE180	Klebsiella variicola	University of Maryland Medical Center	6,381,124	963,549,724	173	58	5,574,578	57.35	185,995	JABKRL000000000	SRR11819344
CRE184	Klebsiella variicola	University of Maryland Medical Center	4,741,274	715,932,374	127	42	5,633,553	57.31	310,605	JABKRK000000000	SRR11819343
CRE607	Klebsiella quasipneumoniae	UCLA Harborview	6,227,888	940,411,088	180	48	5,211,910	57.63	264,029	JABKKR000000000	SRR11819342

### Data availability.

All data have been made publicly available and are deposited under the accession numbers listed in Table 1.
